# Dark septate endophyte *Exophiala pisciphila* promotes maize growth and alleviates cadmium toxicity

**DOI:** 10.3389/fmicb.2023.1165131

**Published:** 2023-04-11

**Authors:** Lei Wang, Zuran Li, Guangqun Zhang, Xinran Liang, Linyan Hu, Yuan Li, Yongmei He, Fangdong Zhan

**Affiliations:** ^1^College of Resources and Environment, Yunnan Agricultural University, Kunming, China; ^2^College of Horticulture and Landscape, Yunnan Agricultural University, Kunming, China

**Keywords:** cadmium forms, phytohormone, root morphology, glutathione metabolism, lignin

## Abstract

Dark septate endophytes (DSE) are typical root endophytes with the ability to enhance plant growth and tolerance to heavy metals, but the underlying mechanisms are unclear. Here, the physiological and molecular mechanisms of a DSE strain, *Exophiala pisciphila*, in mitigating cadmium (Cd, 20 mg/kg) toxicity in maize were investigated. Our results showed, under Cd stress, *E. pisciphila* inoculation enhanced the biomass of maize and reduced both inorganic and soluble forms of Cd (high toxicity) by 52.6% in maize leaves, which may be potentially contributing to Cd toxicity mitigation. Besides, *E. pisciphila* inoculation significantly affected the expression of genes involved in the signal transduction and polar transport of phytohormone, and then affected abscisic acid (ABA) and indole-3-acetic acid (IAA) contents in maize roots, which was the main reason for promoting maize growth. In addition, *E. pisciphila* also made a 27% increase in lignin content by regulating the expression of genes involved in the synthesis of it, which was beneficial to hinder the transport of Cd. In addition, *E. pisciphila* inoculation also activated glutathione metabolism by the up-regulation of genes related to glutathione S-transferase. This study helps to elucidate the functions of *E. pisciphila* under Cd stress, sheds light on the mechanism of detoxifying Cd and provides new insights into the protection of crops from heavy metals.

## 1. Introduction

The heavy metal contamination of soils is a pressing issue worldwide (Marrugo-Negrete et al., 2017; Yang et al., 2018; Han et al., 2020; Qin et al., 2021). The continued increase of heavy metal levels in the soil system leads to toxicity symptoms and inhibits plant growth directly or indirectly (Jaiswal et al., 2018; Ghori et al., 2019). As a typical heavy metal, cadmium (Cd) has attracted particular concern as it is highly toxic to most organisms (Liu et al., 2018; Yang et al., 2018; Wang et al., 2021a). The rapid development of the chemical industry has exacerbated Cd pollution in the soil (Sodango et al., 2018; Zhao et al., 2019; Wang et al., 2021b). Notably, increasing amounts of Cd have entered arable soils with fertilization and wastewater irrigation (Rezapour et al., 2019; Hou, D. et al., 2020; Fu et al., 2021). Once Cd enters the arable soils, it is readily absorbed by food crops (primary producers) due to its high-water solubility, thereby causing toxicity to humans through the food chain (McLaughlin et al., 1999; Nkwunonwo et al., 2020; Suhani et al., 2021). Therefore, it is necessary to take ecological security and sustainable development approaches to reduce the accumulation of Cd in food crops.

Most plants establish symbiotic relationships with microbes in natural ecosystems (He et al., 2020; Su et al., 2021). Plant responses to environmental stresses induced by microbial symbionts have received increasing attention in recent years (Shen et al., 2020; Riaz et al., 2021; Su et al., 2021). Studies have found the association between plants and their rhizosphere microbes, particularly root-associated endophytic microbes, which reside in the internal tissues of plants, and may have positive effects on plant growth and improve the tolerance of plants to stressful environments (Rodriguez et al., 2009; Bedini et al., 2018; Zhan et al., 2018; White et al., 2019). Therefore, the endophytic microbe is considered an efficient strategy for the remediation of contaminated plants. Dark septate endophytes (DSEs) are well-known for dematiaceous septate hyphae and melanized microsclerotia, which are one of the most studied groups of root fungal endophytes (Jumpponen and Trappe, 1998). DSEs are ubiquitous colonists of plant roots in a wide range of terrestrial ecosystems and frequently distributed in stressful environments, particularly in heavy metal-polluted soils (Newsham, 2011; He et al., 2017; Hou, L. et al., 2020; Su et al., 2021).

Accumulating evidence supports that DSEs can influence the metal tolerance of plants and improve the resistance of plants to heavy metal stress (Shen et al., 2020; Wu et al., 2020; Hou, L. et al., 2020). It was demonstrated that DSE inoculation activates the glutathione (GSH) metabolism and protects the plants against heavy metal stress, because of the significant enhancement of glutathione reductase (GR) and GSH (Zhan et al., 2017). Moreover, DSE inoculation can alter the contents of various phytohormones, such as indoleacetic acid (IAA) and abscisic acid (ABA) and improve plant growth by promoting plant nutrient uptake (He et al., 2017; Wu et al., 2020; Xu et al., 2020). DSEs are closely associated with the roots of many host plants. It colonizes the root cortex of the host plants, induces changes in root traits, and promotes root growth of the host plant (e.g., root length, surface area, and biomass) (He et al., 2017). DSE inoculation can also contribute to impeding Cd transport from roots to shoots, decrease the Cd content in shoots and retain Cd in the DSE-inoculated roots (Hou, L. et al., 2020; Su et al., 2021; Xiao et al., 2021). For example, DSE has been reported to increase the root mass density (root mass per root volume), favoring the mineral nutrients storage of roots, and possibly contributing to the storage of Cd ions in the roots (Kramer-Walter et al., 2016). In addition, fungal melanin in DSE is thought to be involved in enhancing the structural rigidity of cell walls, which may contribute to the tolerance of fungus to stress (Eisenman and Casadevall, 2012; Berthelot et al., 2017). These outstanding researches have expounded the important role of DSE in improving plant tolerance from different perspectives, but there is no comprehensive investigation of it, and the corresponding molecular mechanism has not yet been elucidated.

In this study, a specific DSE strain with a high resistance to Cd stress, *Exophiala pisciphila* H93 (accession number ACCC32496, China Agricultural Culture Collection), was selected as the model DSE-association to investigate the growth, physiology, and molecular mechanisms of DSE-alleviated Cd stress in maize. The effect of DSE on the biomass, root morphological traits, phytohormone, sulfhydryl compounds, and Cd content of maize planted in Cd-contaminated soils was investigated. In addition, we applied transcriptome sequencing to explore the molecular mechanism underlying Cd detoxification by *E. pisciphila*. We focus on: (i) how *E. pisciphila* colonization reduces Cd toxicity to maize seedlings by altering morphological and physiological traits: (ii) how *E. pisciphila* colonization enhances plant tolerance of maize seedlings to Cd stress; and (iii) the transcriptomic mechanism of *E. pisciphila* associated with Cd detoxification in maize.

## 2. Materials and methods

### 2.1. Experimental design

#### 2.1.1. Materials preparation

*Exophiala pisciphila* was isolated from the roots of a gramineous species (*Arundinella bengalensis*) growing naturally at an abandoned mining area in Huize County, Yunnan Province, China (103°36′ E, 26°55′ N) (Li et al., 2011). This fungus was preserved in the China Agricultural Culture Collection as accession No. ACCC32496. The *E. pisciphila* strain was cultivated in the potato dextrose agar (PDA) medium (potato 200 g, dextrose 20 g, agar 18 g, and water 1,000 mL) at 28°C for 2 weeks for its activation. A main locally cultivated maize variety, Huidan No. 4, was chosen as the host plant, which was a variety with high Cd tolerance and low Cd accumulation screened by the research group (Chen et al., 2014). The seeds were soaked in 10% sodium hypochlorite for 2 min, and 75% ethanol for 1 min for sterilization, then rinsed 3 times with sterile water and placed in a petri dish with water agar medium (agar 8 g L^−1^) for aseptic germination (25°C for 3 days).

#### 2.1.2. Preparation of inoculated/non-inoculated maize seedlings

Cylinder glass bottles (6.5 cm in diameter, 40 cm in height) containing 0.4 kg quartz sand (autoclaved at 121°C for 2 h) and 40 mL 50% Hoagland medium for fungal inoculation. For the treatment with *E. pisciphila* inoculation, 10 g PDA containing *E. pisciphila* and 2 maize seedlings were transferred to the bottles. During the growth of the maize seedlings, the roots attached to the *E. pisciphila* colonies, *E. pisciphila* mycelium subsequently infected the roots. For the treatment without *E. pisciphila* inoculation, 10 g PDA without *E. pisciphila* colonies and 2 maize seedlings were used. All the bottles were covered with sterile AeraSeal films (150 × 150 mm) (Mycomebio Bio-medical Science Technology Center, China) and cultivated in a glasshouse with a 10 h photoperiod (1,000–8,000 lx) at 28°C/15°C (daytime /nighttime) and 75% humidity for 14 days.

After 14 days, the *E. pisciphila*-inoculated seedlings were checked for DSE colonization by observing the presence of microsclerotia or hyphae in the root cells with a compound microscope (Olympus-BX51, Japan). Five 0.5 cm root fragments for each seedling were randomly collected and washed with deionized water, softened in a water bath with 10% (w/v) KOH at 90°C for 2 h and then stained with 0.5% acid fuchsin (Berch and Kendrick, 1982). The stained roots were pressed onto slides and observed under a compound light microscope (Olympus-BX51, 200 magnification) to determine the fungal colonization intensity with the magnified intersection method (McGonigle et al., 1990).

#### 2.1.3. Greenhouse pot cultivation

Quartz sand (0.4 kg), 50% Hoagland medium (40 mL), and PDA (10 g) with/without *E. pisciphila* colonies were used as the culture substrate filled into cylinder glass bottles, and the Cd ^2+^ (CdCl_2_·2.5H_2_O was added to the Hoagland medium to achieve a Cd ^2+^ concentration of 200 mg/L, resulting Cd ^2+^ concentration in the quartz sand was 20 mg/kg) was supplemented to half of the bottles. Based on our previous study, under 20 mg/kg Cd stress, *E. pisciphila* colonization in maize roots significantly increased maize (Huidan No. 4) biomass, plant height and Cd accumulation in the roots (He et al., 2017; Xiao et al., 2021). The four treatments were the Control (non-inoculated *E. pisciphila*, 0 mg/kg Cd ^2+^), DSE treatment (inoculated *E. pisciphila*, 0 mg/kg Cd ^2+^), Cd treatment (non-inoculated *E. pisciphila*, 20 mg/kg Cd ^2+^), Cd + DSE treatment (inoculated *E. pisciphila*, 20 mg/kg Cd ^2+^), respectively. Two non-inoculated maize seedlings of similar sizes were carefully planted in each of the glass bottles of the Control and Cd treatments, while DSE-inoculated maize seedlings were used for the DSE and Cd + DSE treatments, with 6 replicates for each treatment (half replicates were used to measure biomass, root morphology, and the other half were used for tolerance physiology and transcriptome). All inoculated treatments were successfully colonized by *E. pisciphila,* the average colonization intensity of DSE and Cd + DSE treatment was 34.80 and 42.56%, respectively, but DSE structures were not observed in noninoculated treatments. All glass bottles were placed in a glasshouse with a day temperature 28°C and night temperature 15°C for 28 days, irrigated the maize seedlings with deionized water until the plants were harvested.

### 2.2. Indicator determination

#### 2.2.1. Biomass, root morphological traits and anatomical structure

The maize seedlings were divided into shoots and roots to determine the biomass and morphological traits. The roots were scanned with a scanner (Perfection V700 Photo) and analyzed the root morphological traits with the WinRHIZO Pro root system analyzer. The shoots and roots were dried at 70°C for 72 h to determine the biomass. In order to observe the root structure, root samples were prepared following the method used by Wu et al. (2018) with modifications. Root apical segments (8 mm from the root apex) were fixed in a Formalin-Aceto-Alcohol (FAA) solution (formalin: acetic acid: 70% alcohol = 1:1:16) for 24 h (Yue et al., 2019). Subsequently, the samples were dehydrated in ethanol and embedded in paraffin. Cross-sections (thick 8–12 μm) were sectioned with a Rotary Microtome (RM 2016, Leica, Germany), and stained with water-soluble safranin and fast green to detect the xylem (Wu et al., 2018). The sections were observed with a microscope (DM2000 LED, Leica, Germany), and documented using Motic Images analyzer (Motic China Group Co., Ltd.).

#### 2.2.2. Cadmium content and chemical forms

The dried leaves and roots (0.1 g) of maize seedling were digested with a mixture of HNO_3_ and HClO_4_ (v/v 3:1) and diluted into a volumetric flask (50 mL) using 0.2% HNO_3_ to measure the content of Cd by an Atomic Absorption Spectrometer (TAS-990, Beijing Puxi, China) (Zhan et al., 2015). Three replicates per treatment.

According to the methods mentioned in Luo et al. (2017) with minor modifications, 80% ethanol, 1 mol/L NaCl and 2% acetic acid were used to extract ethanol-extracted state Cd ions (F_E_-Cd), sodium chloride state Cd ions (F_NaCl_-Cd), acetic acid state Cd ions (F_HAc_-Cd), respectively. Fresh maize sample (0.5 g) was ground into homogenate in extraction solution, then transferred to a 50 mL centrifuge tube [diluted to 1:50 (w/v)] and shaken at 25°C for 22 h. The first supernatant solution was obtained by centrifuging the homogenate at 5,000 *g* for 10 min. The sedimentation was resuspended in extraction solution and shaken for 1 h at 25°C, centrifuged at 5,000 *g* for 10 min, then the supernatants of two times suspensions and centrifugation steps were combined to obtain different chemical forms Cd. Supernatant solutions were evaporated on an electric plate at 70°C to a constant weight and digested with an acid oxidative mixture of HNO_3_/HClO_4_ (3:1, v/v) at 145°C, then determined the concentrations of Cd associated with different chemical forms by an Atomic Absorption Spectrometer (TAS-990, Beijing Puxi, China).

#### 2.2.3. Phytohormone

Abscisic acid (ABA) and indole-3-acetic acid (IAA) contents were estimated according to double-antibody method with ELISA kits (Shanghai Huyu Biotechnology Co. Ltd., Shanghai, China) (Hedden, 1993), each sample was examined in triplicate Approximately 0.5 g of roots were ground in a mortar with 10 mL phosphate buffer solution at 4°C. Sample solution (10 μL) was added to the specificity antibody plate (40 μL of 0.15 M phosphate buffer solution per well), and conjugate reagent (50 μL with HRP labeled) was added to each well. Then the color-developing agent was added and stored in the dark for 10 min. Finally, the absorbance was measured at 450 nm after adding the stop solution (H_2_SO_4_) (Chen, X. et al., 2017).

#### 2.2.4. Lignin contents and key enzymes of lignin synthesis

Root samples (0.1 g) from each treatment were used to test the lignin content, 4-coumarate CoA ligase (4CL), cinnamyl-alcohol dehydrogenase (CAD), and peroxidase (POD) activities, determined using commercial kits (Suzhou Grace Bio-technology Co. Ltd., Suzhou, China) according to the previous methods (Cheng et al., 2020). The lignin was determined by the acetylation method, the acetylated lignin had a characteristic absorption peak at 280 nm, and the absorbance value at 280 nm was recorded to calculate the lignin content (Fan et al., 2021). 4CL can catalyze 4-coumarate and CoA to generate 4-coumarate CoA, and the 4CL activity can be reflected by measuring the 4-coumarate CoA generation rate at 333 nm. CAD can catalyze Cinnamyl alcohol to generate Cinnamic aldehyde, and then react with a specific chromogen, and calculate the CAD enzyme activity by detecting the increased rate of colored substances. Under the catalysis of peroxidase, H_2_O_2_ oxidizes specific substrates with maximum light absorption at 470 nm, and the POD activity is determined by measuring the change of absorbance at 470 nm.

#### 2.2.5. Sulfhydryl compounds

The homogenate was centrifuged at 10,000 × *g* at 4°C for 20 min to obtain a supernatant. The glutathione synthetase (GSS), γ-glutamyl cysteine synthetase (γ-GCS), glutathione reductase (GR) and glutathione (GSH) contents were assessed using the methods described in the commercial assay kits from Nanjing Jiancheng Bioengineering Institute (Nanjing, China) according to the previous methods (Zhan et al., 2017). The γ-GCS and GR assay kit was designed using principles described by Seelig and Meister (1985) and Foster and Hess (1980), respectively. NADH and NADPH oxidation were assessed by measuring the decrease in absorbance at 340 nm at 37°C. The activity of γ-GCS was determined as the amount of enzyme necessary for the consumption of 1 μ mol of NADH per minute, while the activity of GR was defined as the oxidation of 1 nmol of NADPH per minute (Zhan et al., 2017). The content of glutathione (GSH) was determined using a colorimetric microplate assay following the instructions provided by Nanjing Jiancheng Bioengineering Institute. After washing the plant tissue with pre-cooled PBS, the supernatant obtained from centrifugation was used to measure the absorbance values at 405 nm, which were then used to determine the GSH content. GSS activities were determined by measuring the ATP-dependent formation of -glutamyl hydroxamate from L-glutamate and hydroxylamine in the supernatant. GSS activity was defined as nmol of -glutamyl hydroxamate produced per second with absorbance measured at 550 nm at 37°C (Yajun et al., 2008).

### 2.3. Transcriptome sequencing

Three root sub-samples (0.5 g) for RNA extraction were obtained from well-growing roots of maize seedling, each treatment was examined in triplicate. Total RNA was extracted by Trizol-extraction methods with TRIzol RNA reagent (Invitrogen Inc., United States). The RNA concentration and integrity were determined and assessed using the Qubit 2.0 Fluorometer (Thermo Fisher Scientific Inc., United States) and Agilent 2100 Bioanalyzer Instrument (Agilent Technologies, Inc., United States, only RNA Integrity Number ≥ 7 was used for RNA-Seq analysis). Genes with false discovery rate (FDR) < 0.05 and absolute fold change ≥2 were defined as differentially expressed genes (DEGs), transcripts were considered significantly differentially expressed. For details, please refer to the section 1 of Supplementary material. Four genes were randomly selected to confirm the accuracy of RNA-Seq through qRT-PCR. There was a significant correlation between the RNA-Seq and qPCR data (*p* < 0.001; Supplementary Figure S1).

### 2.4. Statistical analyses

All data analyses were performed in R version 3.6.1 (Team, 2020) and SPSS 25.0 (SPSS, Inc.), the data were log-transformed when needed. The *t*-test was performed in SPSS 25.0 to test the differences in Cd accumulation and chemical forms between Cd and Cd + DSE treatments. One-way analysis of variance was used to test the responses of maize traits to DSE inoculation and Cd stress (“multcomp” package, Tukey’s HSD, checking for homogeneity of variances with Levene’s test). Plots were generated using GraphPad Prism 8.0. DEGs for each pairwise comparison was analyzed with the “edgeR” package. Visualization of GO terms were generated by using the “REVIGO” web service.

## 3. Results

### 3.1. Biomass and root morphological traits of maize seedlings

**Figure 1:**
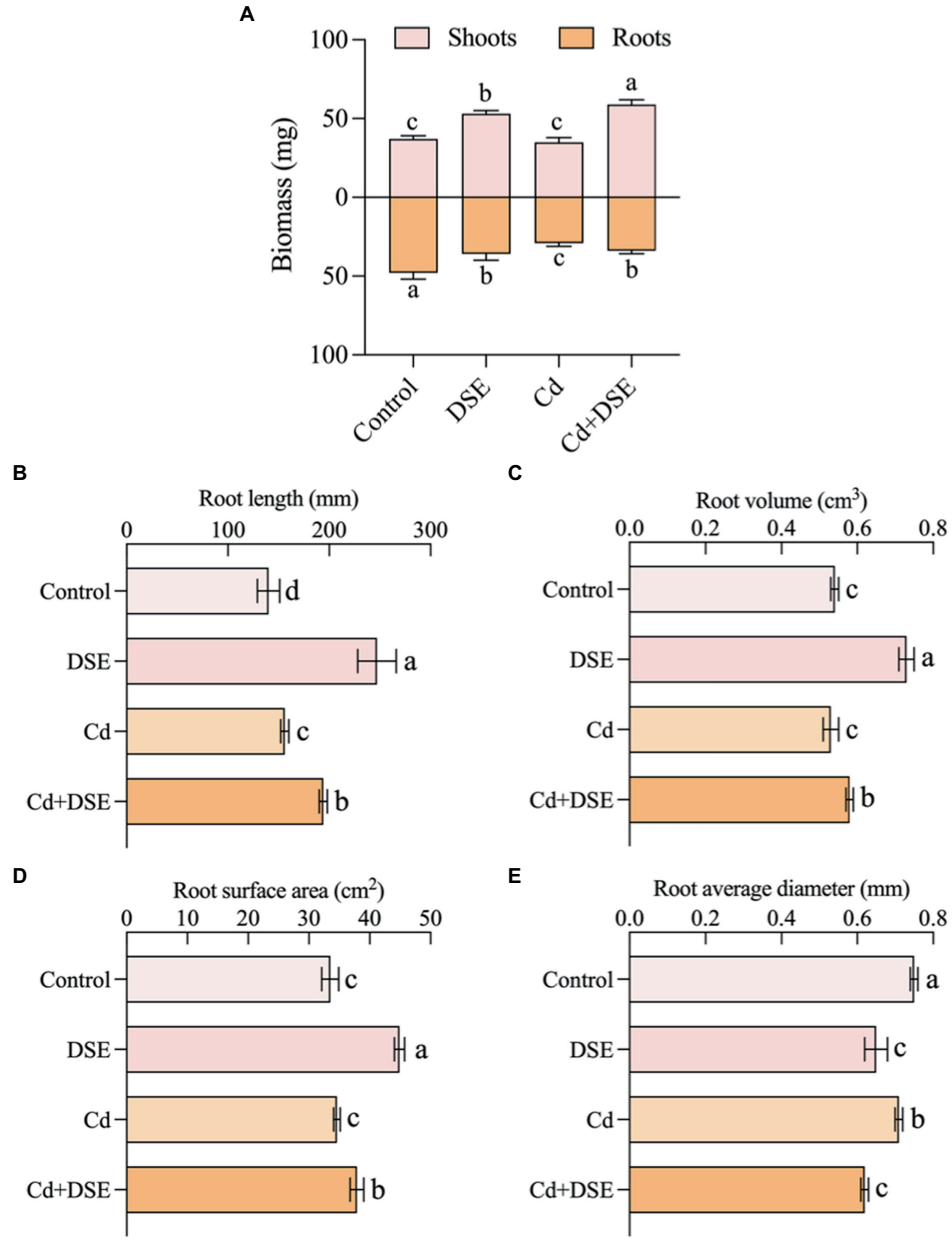
Responses of shoots and roots biomass **(A)** and morphological traits, including root length **(B)**, root volume **(C)**, root surface area **(D)** and root average diameter **(E)** of maize to *E. pisciphila* inoculation and Cd stress. Data were mean values (*n* = 3) and error bars represent the standard deviation. Different letters indicate signiﬁcant differences between treatments.

In this study, *E. pisciphila* inoculation (without Cd stress) induced a significant increase in the shoot biomass of maize seedlings by 43.2%, relative to the control. The shoot biomass under Cd stress treatment (non-inoculated *E. pisciphila*) did not demonstrate any significant differences from the control, however, the root biomass exhibited significant decreases by 39.3%. In addition, Cd stress inoculated with *E. pisciphila* (Cd + DSE treatment) significantly increased shoot and root biomass of maize seedlings by 68.8 and 16.8%, respectively, relative to the biomass under Cd stress (Figure 1A). In addition, the root length, volume, and surface area of DSE treatment (*E. pisciphila* inoculation only) signiﬁcantly increased by 76.4%, 35.2% and 34.0% relative to the control, respectively, but the average diameter exhibited a signiﬁcant decrease of 13.3% on average (Figures 1B–E). Cd stress with *E. pisciphila* inoculation resulted in a signiﬁcant increase in the root length, volume, and surface area relative to the Cd stress treatment by 24.4%, 9.5%, and 9.5%, respectively. However, there was a signiﬁcant decrease in the average diameter by an average of 12.7% (Figures 1B–E).

### 3.2. Cd content and chemical forms of maize seedlings

**Figure 2:**
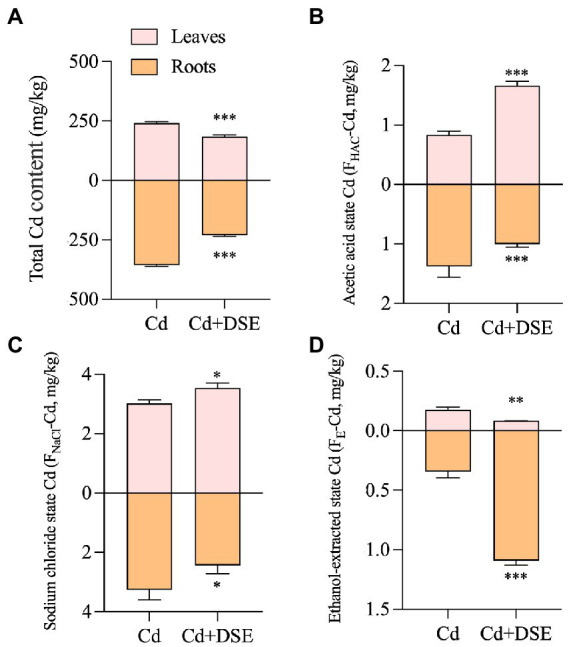
Responses of Cd accumulation **(A)** and chemical forms, including acetic acid state Cd **(B)**, sodium chloride state Cd **(C)** and ethanol-extracted state Cd **(D)** of maize to *E. pisciphila* inoculation. Data were mean values (*n* = 3) and error bars represent the standard deviation. “NS,” “*,” “**,” and “***” mean no significance, *p* < 0.05, *p* < 0.01, and *p* < 0.001, respectively.

As the *E. pisciphila* was inoculated (Cd + DSE treatment), the Cd contents of both the leaves and roots of the maize significantly decreased by 23.6 and 35.3% relative to the Cd stress (Cd treatment, Figure 2A). Compared with the Cd treatment, the Cd stress with *E. pisciphila* inoculation (Cd + DSE treatment) significantly increased the F_HAc_-Cd and F_NaCl_-Cd contents of the maize leaves by 98.8 and 14.5%, respectively, but significantly reduced the F_E_-Cd contents by 52.6% (Figures 2B–D).

### 3.3. Phytohormone contents, lignin content, sulfhydryl compounds and related enzyme activities of maize seedlings

**Figure 3:**
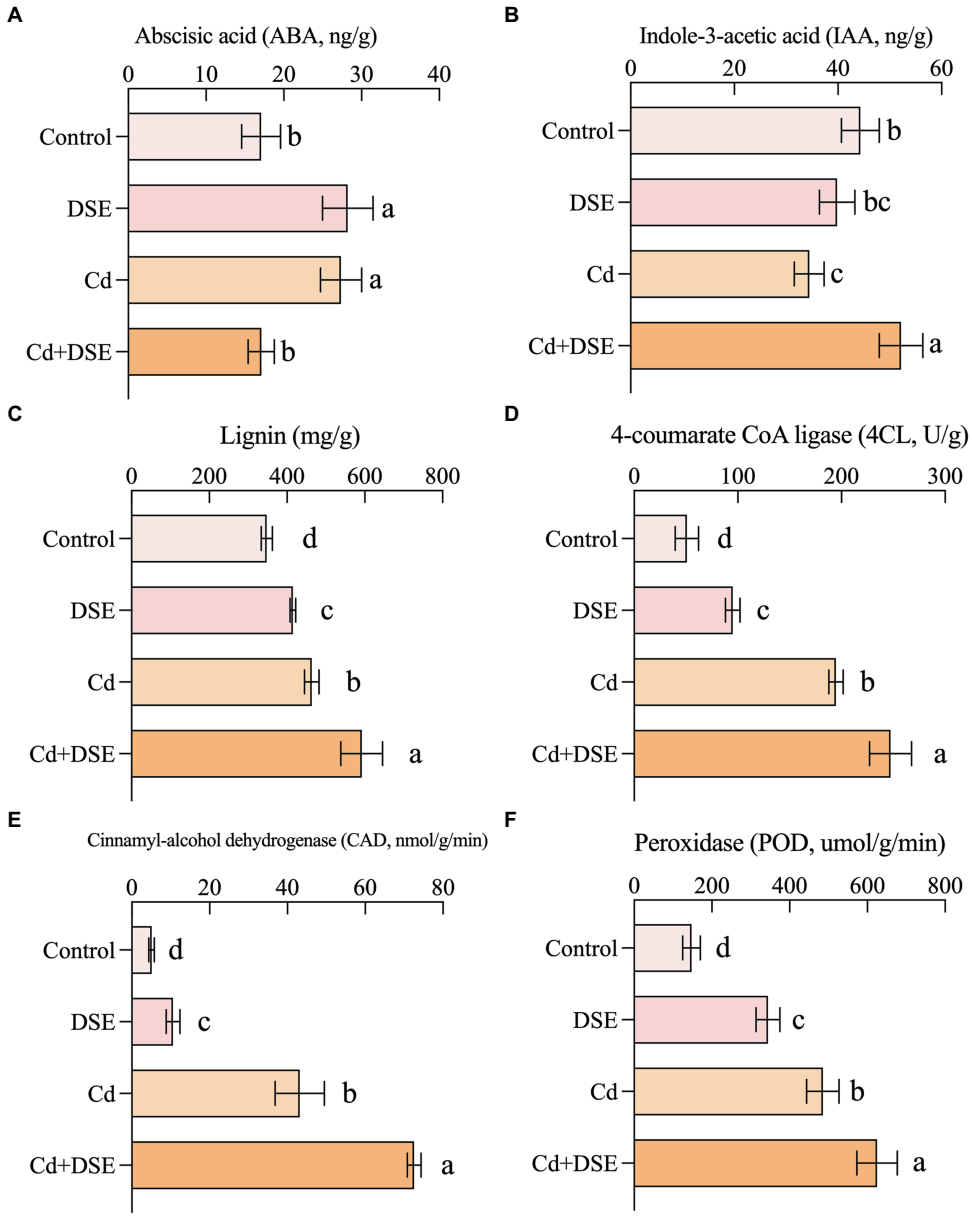
Responses of phytohormone and lignin contents and related enzyme activities of maize roots to *E. pisciphila* inoculation and Cd stress. **A**: abscisic acid, **B**: Indole-3-acetic acid, **C**: Lignin; **D**: 4-coumarate CoA ligase, **E**: Cinnamyl-alcohol dehydrogenase, **F**: Peroxidase. Data were mean values (*n* = 3) and error bars represent the standard deviation. Different letters indicate signiﬁcant differences between treatments.

**Figure 4:**
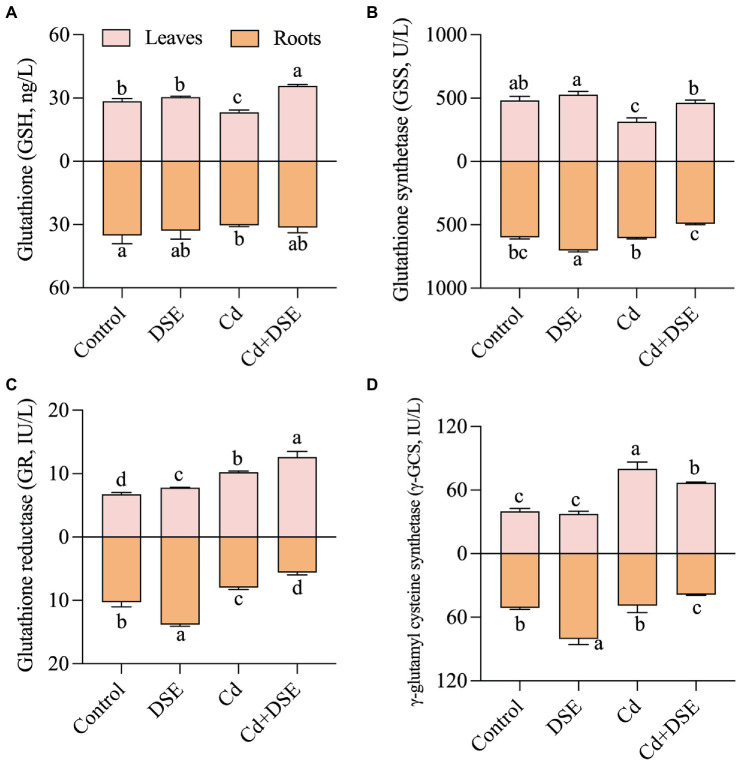
Responses of sulfhydryl compounds of maize leaves and roots to *E. pisciphila* inoculation and Cd stress. **A**: glutathione, **B**: glutathione synthetase, **C**: glutathione reductase; **D**: γ-glutamyl cysteine synthetase. Data were mean values (*n* = 3) and error bars represent the standard deviation. Different letters indicate signiﬁcant differences between treatments.

In the current study, with DSE inoculation (without Cd stress), the content of ABA showed a significant increase relative to the control by 65.2% (Figure 3A). The ABA content significant increased by 60.1%, while IAA content significantly decreased by 22.3% with the Cd treatment, relative to the control. Moreover, compared with the Cd stress, DSE inoculation under Cd stress (Cd + DSE treatment) resulted in a significant increase in the IAA content, with an average increase of 51.5%, whereas ABA content was significantly decreased by 37.4% (Figures 3A,B). Compared with the control, Cd treatment, DSE treatment and Cd stress with DSE inoculation (Cd + DSE) treatments resulted in a significant increase in lignin content, 4CL, CAD, and POD activities (Figures 3C–F). Moreover, compared with the Cd treatment, DSE inoculation under Cd stress (Cd + DSE) resulted in a significant increase in the lignin content by 27.7%, as well as the 4CL, CAD and POD activities by 27.1, 68.2 and 28.8%. In the present study, we found that the GR and γ-GCS activities in the maize leaves increased significantly under Cd treatment (Figures 4C,D). Moreover, DSE inoculation resulted in a significant increase in the GSH content, GSS, and GR activities in the leaves of maize under Cd stress (Cd + DSE, Figures 4A–C).

### 3.4. Transcriptome sequencing

**Figure 5:**
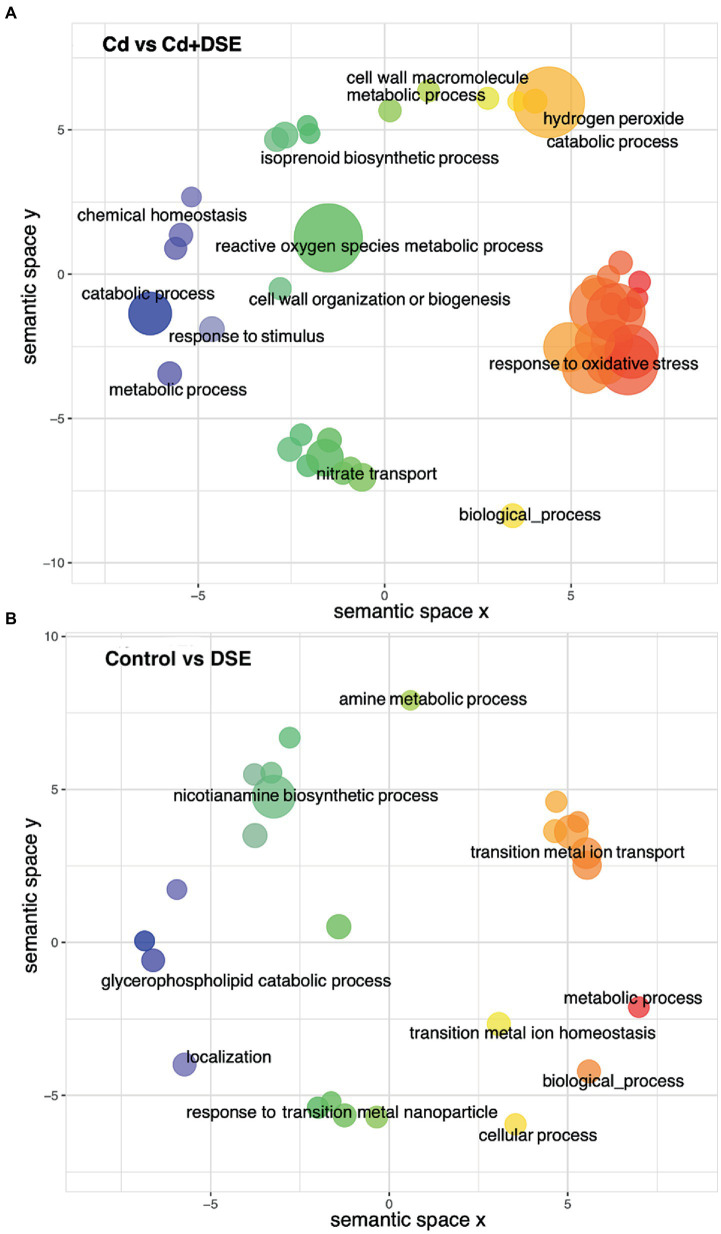
Graphical depiction of enriched GO-BPs in **(A)** Cd vs. Cd + DSE and **(B)** Control vs. DSE (FDR < 0.05). GO-BP terms are colored by semantic similarity to other GO terms and bubble size reflects the abs_log10_pvalue of the GO-term in the Fisher test. The two-dimensional semantic space was generated by the REVIGO web service.

**Figure 6:**
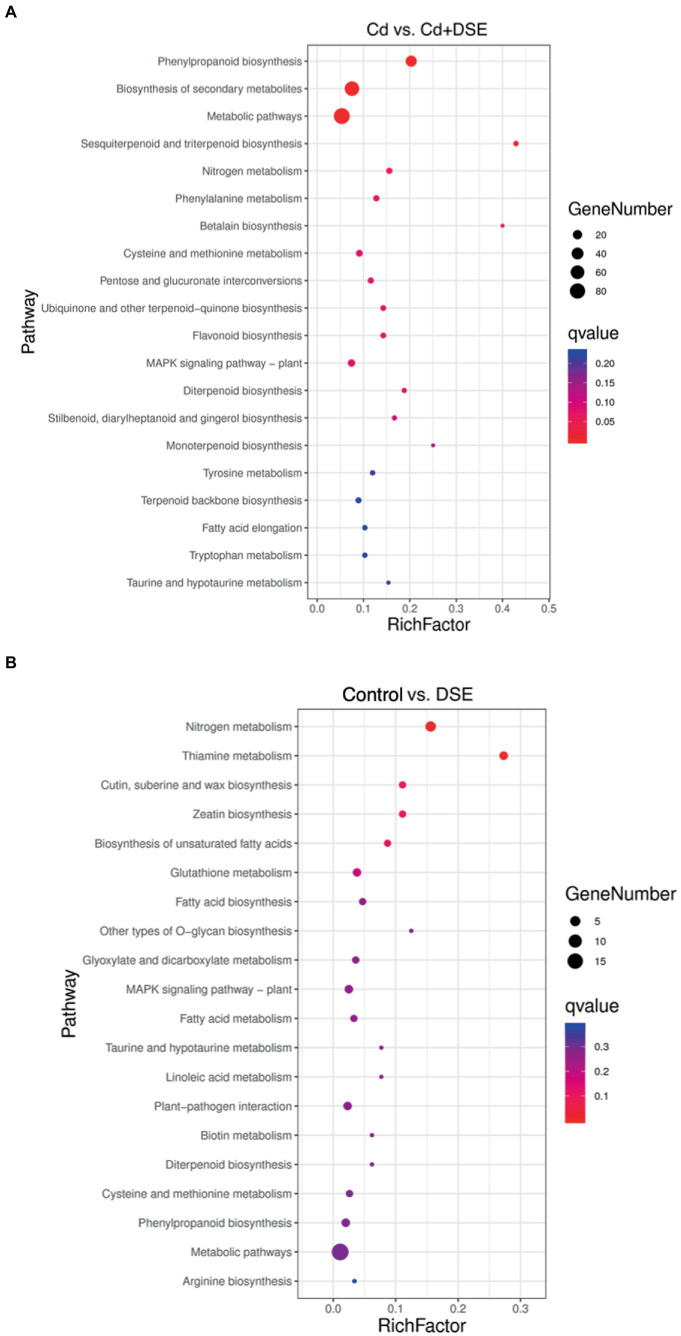
Top 20 of KEGG enrichment pathway in **(A)** Cd vs. Cd + DSE and **(B)** Control vs. DSE treatments.

DSE treatment resulted in differences in 433 differentially expressed genes (DEGs). More specifically, 287 DEGs were up-regulated and 146 DEGs were down-regulated, relative to the control. In addition, compared with the Cd stress treatment, 948 DEGs were different after DSE inoculation, among which 529 DEGs were up-regulated and 419 DEGs were down-regulated (Supplementary Table S1). A higher number of GO enriched biological process (BPs) was recognized in the ‘Cd versus Cd + DSE.’ Among these, the most significant BPs (FDR < 0.01) were the hydrogen peroxide catabolic process, the hydrogen peroxide metabolic process, the reactive oxygen species metabolic process, the oxidation–reduction process and response to oxidative stress (FDR < 0.01, Figure 5A). GO enriched BPs in ‘Control versus DSE’ included the nicotianamine metabolic process, the nicotianamine biosynthetic process, the tricarboxylic acid biosynthetic process, transmembrane transport and the oxidation–reduction process (FDR < 0.01, Figure 5B). Among the enriched KEGG pathways of ‘Cd versus Cd + DSE’, the most significant pathways were identified as phenylpropanoid biosynthesis, biosynthesis of secondary metabolites, metabolic pathways, sesquiterpenoid and triterpenoid biosynthesis, and the nitrogen metabolism (Figure 6A). The enriched KEGG pathways of ‘Control versus DSE’ included the nitrogen metabolism, thiamine metabolism, cutin, suberine and wax biosynthesis, zeatin biosynthesis and biosynthesis of unsaturated fatty acids (Figure 6B).

**Figure 7:**
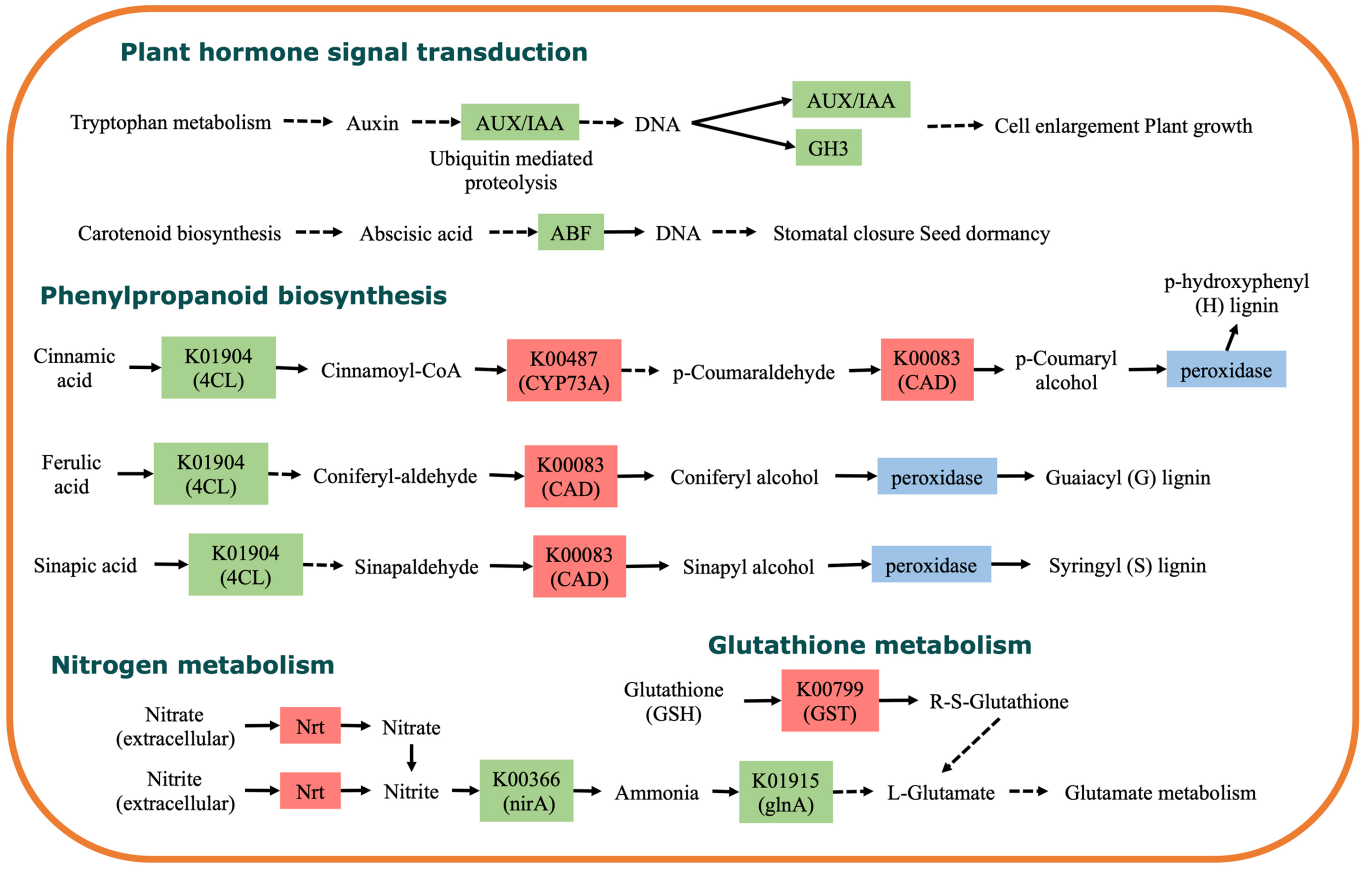
KEGG pathway enriched by differentially expressed genes between Cd and Cd + DSE treatments. Colors indicate significant differential expression, red represents up-regulation, green represents down-regulation, and blue represents both up-regulation and down-regulation.

The transcriptomic analysis revealed that the expression of early auxin-responsive genes such as *IAA24* and *GH3.6* in the plant hormone signal transduction pathway were significantly reduced under Cd stress with DSE inoculation compared with Cd stress (Figure 7 and Supplementary Table S3). Moreover, DSE inoculation significantly down-regulated the gene expression in *E3 ubiquitin-protein ligase* (regulate the ubiquitination of IAA) under Cd stress. DSE inoculation under Cd stress significantly up-regulated the expression of ABA-responsive genes (*Abscisic acid stress ripening*), as well as ABA-related protein genes (*Pathogenesis-related protein*) (Figure 7 and Supplementary Table S4), which regulated ABA content in maize. In addition, transcriptomic analysis revealed that DSE inoculation under Cd stress significantly affected the synthesis of p-hydroxyphenyl (H), guaiacyl (G), and syringyl (S) lignin in the phenylpropanoid biosynthesis pathway (Figure 7). DSE inoculation under Cd stress also led to the up-regulation of glutathione S-transferase related genes in the Glutathione metabolism pathway (Figure 7).

## 4. Discussion

In the present study, maize seedlings were subjected to Cd stress, and those treated with *E. pisciphila* inoculation exhibited a significant increase in both shoot and root biomass. The results are consistent with those reported by Wu et al. (2020), Su et al. (2021), and Xiao et al. (2021), who found that the shoot and root biomass of maize, rice, and blueberry seedlings increased after inoculation with DSE. In addition, with *E. pisciphila* inoculation, the root length, surface area, and volume of the maize seedlings were significantly increased regardless of the presence of Cd stress. Hou, L. et al. (2020) also reported that DSE inoculation enhanced the root length and surface area of *Medicago sativa*, which facilitated plant growth and improved Cd tolerance. Ban et al. (2017) found that DSE inoculation increased the root length of maize seedlings subjected to stress induced by exposure to different Pb concentrations, indicating that DSE inoculation can improve the growth of maize roots. Thus, the research indicates that *E. pisciphila* inoculation affects the root morphological traits of maize seedlings in such a way that is conducive to plant growth.

In addition to the above results, we also observed a significant shift in the chemical forms of Cd in the *E. pisciphila*-colonized maize. We argue that these chemical forms of Cd are closely related to their biological toxicity, as they determine their reactivity and solubility. Among the various chemical forms of Cd, the Cd-phosphate complex extracted by 2% acetic acid (F_HAc_-Cd) is important for plant tolerance to Cd due to its insolubility, low mobility, and low toxicity (Qiu et al., 2011). Compared with Cd stress without inoculation, Cd stress with DSE inoculation significantly increased the F_HAc_-Cd and F_NaCl_-Cd contents of the maize leaves but reduced their F_E_-Cd contents. Similarly, studies on *Poa pratensis* and *Festuca arundinacea* have shown that an increased level of undissolved Cd-phosphate complexes (extracted by 2% acetic acid, F_HAc_-Cd) favored Cd tolerance (Xu and Wang, 2013). The inorganic, water-soluble form of Cd (FE-Cd, which can be extracted with 80% ethanol) has a greater negative effect on plants than the effect caused by Cd complexed with phosphate and undissolved (FHAc-Cd, extracted with 2% acetic acid). Wang et al. (2016) found that DSE increased the amount of inactive Cd in maize and reduced both the soluble and inorganic content of Cd, however, there were no similar effects noted in the maize roots. In this study, *E. pisciphila* inoculation reduced the F_E_-Cd content in maize leaves, which helped to alleviate the toxicity of Cd to plants.

Phytohormones are important regulators of heavy metal tolerance in plants, which enhance plant adaptation to environmental stress by regulating adaptive responses. In the current study, *E. pisciphila* inoculation prior to Cd stress resulted in a significant increase in the IAA content and decreased the ABA content in plant roots compared with the Cd stress without inoculation. This finding is consistent with those reported by He et al. (2017), whereby DSE inoculation significantly increase in the IAA content in maize exposed to a Cd concentration of 20 mg/kg, whereas ABA content significantly decreased under the same treatment. Similarly, Khan and Lee (2013) found that root colonization by endophytes resulted in a significant decrease in the ABA content of *Glycine max* L. under heavy metal stress. Furthermore, increased root volume may be associated with the activation of the IAA in the roots of maize, which is induced by *E. pisciphila* colonization. IAA can effectively stimulate the growth of host plant roots, improve nutrient uptake, and promote plant growth (Khan et al., 2012; Chen, B. et al., 2017). Furthermore, studies have shown that endophytic bacteria can promote plant growth under Cd stress by producing IAA and directly regulating the expression of genes involved in Cd uptake and transport and that IAA may help plants alleviate the toxicity of Cd to cells (Sukumar et al., 2013; Chen, B. et al., 2017). Therefore, *E. pisciphila* can improve plant growth by regulating the phytohormone contents in response to Cd stress.

As the outer barrier of plants, the Casparian strip functions as a physiological fence and valve, which can control the entry of water and mineral ions into vascular tissues, protect against abiotic stress, and defend against the infiltration of toxic compounds. Lignin, which is the main component of the Casparian strip, was found to be significantly increased under Cd stress both with and without *E. pisciphila* inoculation. Líška et al. (2016) demonstrated that Cd exposure resulted in the asymmetric development of the exodermis and endodermis structures of maize roots, while the cell wall of the exodermis was significantly thickened to reduce the uptake and transport of harmful ions by the roots. Moreover, compared with maize subjected to Cd stress, those treated with *E. pisciphila* inoculation prior to Cd stress exhibited a significant increase in the lignin content as well as the activities of 4CL, CAD and POD, which were positively associated with lignin synthesis. Additionally, lignin is reported to be an ideal site for metal ion binding *via* the various functional groups (Guo et al., 2008). Our results showed, under Cd stress of DSE-inoculated maize promoted the Cd tolerance of host, including the Cd compartmentation by Casparian strip. We argue that *E. pisciphila* can significantly increase the lignin content, inhibit the migration of Cd from the cortex into the central column, and hinder the transport of Cd.

Sulfhydryl compounds play an important role in the response of plants to heavy metal stress. Among the various heavy metal tolerance mechanisms employed by plants, sulfhydryl compounds act by chelating heavy metal ions to form low-toxicity products (Wang et al., 2019; Chen et al., 2021). In the present study, under Cd stress, the activities of GR and γ-GCS activities in the maize leaves significantly increased. Similarly, the activity of GR was significantly increased in two mustard cultivars after exposure to Cd stress (Iqbal et al., 2010). Moreover, under Cd stress, the GSH content, GSS activity, and GR activity in the leaves of maize increased significantly following *E. pisciphila* inoculation. Similar to our results, DSE colonization increased the GSH content and enhanced the GR activity of maize under the Cd treatment, while also decreasing the Cd content in maize leaves (Zhan et al., 2017). Pan et al. (2016) also determined the positive effects conferred by endophytic inoculation and observed an increase in the GSH concentration, Cd tolerance, and accumulation of Cd in the roots of *Sedum alfredii*. Studies on tobacco have also shown that endophytes significantly increased the expression of genes related to the GSH metabolism and promoted the retention of Cd in tobacco roots (Hui et al., 2015). These results suggest that sulfhydryl compounds and enzymes respond positively to Cd stress and tolerance.

The transcriptome was analyzed to explore the underlying mechanisms of the above results. Under Cd stress, gene expression related to the early auxin- response and the ubiquitination of IAA were significantly downregulated following *E. pisciphila* inoculation compared to the condition of Cd stress non-inoculation, which are crucial for maintaining IAA homeostasis in plants (Yue et al., 2016; Li et al., 2017). *E. pisciphila* inoculation also significantly upregulated the genetic expression of ABA-responsive and ABA-related proteins, which regulated ABA content in maize. In addition, under Cd stress, *E. pisciphila* inoculation significantly affected the expression of genes related to the synthesis of lignin. This is attributed to the significant changes in the expression of 4CL, CAD, and POD induced by *E. pisciphila*. *E. pisciphila* inoculation under Cd stress also led to the upregulation of glutathione S-transferase-related genes in the glutathione metabolism pathway. This indicates that the phytohormones, lignin content, sulfhydryl compounds and related enzymes may be involved in the promotion of plant growth induced by *E. pisciphila* inoculation.

In this study, the biological function and molecular mechanism of the DSE strain *E. pisciphila* in mitigating Cd toxicity in maize were investigated. The results demonstrated that *E. pisciphila* inoculation induced a significantly upregulated tolerance to Cd, with a significant decrease in phytotoxicity and an increase in maize root and shoot biomass. *E. pisciphila* promoted maize growth by regulating the expression of phytohormone-related genes to affect phytohormone contents in maize roots, alleviating Cd toxicity by regulating the expression of genes related to lignin synthesis and glutathione S-transferase, increasing lignin contents, and activating glutathione metabolism to reduce the levels of highly toxic forms of Cd. The results of this study help to elucidate the mechanism by which *E. pisciphila* colonization enhances heavy metal resistance in plants and provide a basis for improving plant growth performance at the morphological, physiological, toxicological, and transcriptional levels.

## Data availability statement

The datasets presented in this study can be found in online repositories. The names of the repository/repositories and accession number(s) can be found at: https://www.ncbi.nlm.nih.gov/- PRJNA936425.

## Author contributions

FZ and GZ conceived and designed the experiments. GZ, LH, XL, and ZL performed the experiments. LW analyzed the data and wrote the manuscript. FZ, YL, and YH provided editorial advice. All authors read and approved the final manuscript.

## Funding

This study was financially supported by the National Natural Science Foundation of China (No. 41877130), the Key Project of Yunnan Agricultural Foundation (2017FG001-014), and the Reserve Talents Fund for Young and Middle-Aged Academic and Technological Leaders in Yunnan Province (No 202005AC160038).

## Conflict of interest

The authors declare that the research was conducted in the absence of any commercial or financial relationships that could be construed as a potential conflict of interest.

## Publisher’s note

All claims expressed in this article are solely those of the authors and do not necessarily represent those of their affiliated organizations, or those of the publisher, the editors and the reviewers. Any product that may be evaluated in this article, or claim that may be made by its manufacturer, is not guaranteed or endorsed by the publisher.

## Supplementary material

The Supplementary material for this article can be found online at: https://www.frontiersin.org/articles/10.3389/fmicb.2023.1165131/full#supplementary-material
