# Supplementary material for: Dark septate endophyte Exophiala pisciphila promotes maize growth and alleviates cadmium toxicity
Source: Front Microbiol. 2023 Apr 11;14:1165131. doi: 10.3389/fmicb.2023.1165131 (PMC10126344; doi:10.3389/fmicb.2023.1165131)
Supplement: Supplementary file 1 [file Data_Sheet_1.docx]

**Supplementary Material for:**

Dark septate endophyte *Exophiala pisciphila* promotes maize growth and alleviates cadmium toxicity

Lei Wang ^a^, Zuran Li ^b^, Guangqun Zhang ^a^, Xinran Liang ^a^, Linyan Hu ^a^, Yuan Li ^a^, Yongmei He ^a^, Fangdong Zhan ^a*^

^a^ College of Resources and Environment, Yunnan Agricultural University, Kunming 650201, People’s Republic of China

^b^ College of Horticulture and Landscape, Yunnan Agriculture University, Kunming, 650201, People’s Republic of China

*Corresponding author:

Fangdong Zhan

E-mail address: zfd97@ynau.edu.cn

**Section 1** **Transcriptome sequencing and functional annotation**

According to the manufacturer's instructions (Invitrogen, Carlsbard, CA, USA; Illumina, San Diego, CA), RNA purification, reverse transcription, library construction and sequencing were performed at Genedenovo Biotechnology Co., Ltd. (Guangzhou, China).

In the transcript libraries from the roots of maize seedlings treated by *E. pisciphila* and the untreated control, the average number of raw bases (RawData) was 7.59×109 bp, and the average number of high-quality databases after filtering (CleanData) was 7.53×109 bp. More than 92% of the bases in CleanData had a q-value ≥ 30, the bases containing N in single-end reads in CleanData are less than 0.001%, and the GC ratio of sequence bases is above 54%. Raw sequencing data were checked using Fastp software (https://github.com/OpenGene/fastp, Chen et al., 2018), after trimming for quality, transcriptome assembly was conducted using the Trinity software (Haas et al., 2013). The clean reads were aligned to the ribosomal database of *Zea mays* using bowtie2, then the genome-wide comparison analysis was performed using HISAT2.

The expression profiles of different samples at gene level were firstly explored by cluster analysis. There were clear differences between *E. pisciphila* -inoculated and uninoculated samples, where most differentially expressed genes (DEGs) were up-regulated in one treatment and down-regulated in the other, and vice versa. The PCA results showed that PC1 explained 41.90% of the total variance, separating two sample groups: Cd and Cd+DSE groups. Vertical separation along PC2 occurred between DSE and Control samples, with PC2 explaining 30.30% of the total variance.

The cDNA libraries were sequenced on the Illumina sequencing platform (Genedenovo Biotechnology Co., Ltd, Guangzhou, China). The reads from each biological replicate were individually mapped to the assembled transcriptome, and the expression of each transcript was quantified using the expectation-maximization method (Li & Dewey, 2011). Gene functions were annotated in Gene Ontology (GO) (http://www.geneontology.org) databases and the Kyoto Encyclopedia of Genes and Genomes (KEGG) (http://www.genome.jp/kegg/) pathway enrichment analysis.

**References**

Chen, S., Zhou, Y., Chen, Y., and Gu, J. (2018). fastp: an ultra-fast all-in-one FASTQ preprocessor. *Bioinformatics* 34, i884-i890.

Haas, B. J., Papanicolaou, A., Yassour, M., Grabherr, M., Blood, P. D., Bowden, J., et al. (2013). De novo transcript sequence reconstruction from RNA-seq using the Trinity platform for reference generation and analysis. *Nat. protoc.* 8, 1494-1512.

Li, B., and Dewey, C.N. (2011). RSEM: accurate transcript quantification from RNA-Seq data with or without a reference genome. *BMC Bioinform* 12, 1-16.

**Section 2**

**Table S1.** Relative number of differentially expressed genes (DEGs) and up- or down-regulated genes for each sample group.

| Group | DEGs | Up- regulated | Down- regulated |
| --- | --- | --- | --- |
| Control vs DSE | 433 | 287 | 146 |
| Cd vs Cd+DSE | 948 | 529 | 419 |

**Table S2.** Primers used for qRT-PCR.

| Gene ID | 5'--3' |
| --- | --- |
| *Zm*00001d029829 | TTGCCAGCCACTTAGGAGAG |
|  | CCGTTCCCGCTTCACTTTAC |
| *Zm*00001d043244 | CATACACGCTCATCCCCACC |
|  | CTCCGTCTCGTCCGTCTTG |
| *Zm*00001d012887 | CAAGAGCAAGGAAGACGGGA |
|  | TCCGAGACTGAAGCAACCT |
| *Zm*00001d052269 | TTCAACCACACCGACTTCTACT |
|  | TGGCTCCTCCGTCCATTTTG |

**Table S3.** Effects of *E. pisciphila* inoculation under Cd stress on gene expression in indole-3-acetic acid (IAA) signaling pathway in maize roots.

| Gene ID | log2(fc) | Description |
| --- | --- | --- |
| *Zm*00001d018973 | -1.51 | IAA24-auxin-responsive Aux/IAA family member |
| *Zm*00001d043244 | -2.40 | Indole-3-acetic acid-amido synthetase GH3.6 |
| *Zm*00001d020953 | 1.25 | Protein PIN-LIKES 3 |
| *Zm*00001d052269 | 1.00 | Putative auxin efflux carrier |
| *Zm*00001d046893 | -1.10 | Auxin efflux carrier component 2 |
| *Zm*00001d020852 | -1.70 | Cellulose synthase-like protein E1 |
| *Zm*00001d027983 | -1.34 | E3 ubiquitin-protein ligase ATL6 |
| *Zm*00001d052189 | 3.29 | Putative RING zinc finger domain superfamily protein |
| *Zm*00001d050514 | -1.27 | Putative RING zinc finger domain superfamily protein |
| *Zm*00001d038471 | -1.05 | U-box domain-containing protein 16 |
| *Zm*00001d005195 | 7.58 | RING/U-box superfamily protein |
| *Zm*00001d028374 | -1.10 | U-box domain-containing protein 21 |
| *Zm*00001d029829 | 4.03 | U-box domain-containing protein 33 |
| *Zm*00001d004238 | 2.82 | U-box domain-containing protein 33 |
| *Zm*00001d018190 | -1.49 | RING/U-box superfamily protein |
| *Zm*00001d022454 | -4.28 | F-box family protein |
| *Zm*00001d045517 | 1.39 | RING/U-box superfamily protein |
| *Zm*00001d014562 | 1.06 | IAA-amino acid hydrolase ILR1-like 6 |
| *Zm*00001d012887 | -1.27 | IAA-amino acid hydrolase ILR1 |

**Table S4.** Effects of *E. pisciphila* inoculation under Cd stress on gene expression in abscisic acid (ABA) signaling pathway in maize roots.

| Gene ID | log2(fc) | Description |
| --- | --- | --- |
| *Zm*00001d025401 | 1.90 | Abscisic acid stress ripening5 |
| Zm00001d016760 | 2.85 | Abscisic acid stress ripening6 |
| *Zm*00001d040785 | 3.34 | Abscisic stress-ripening protein 1 |
| *Zm*00001d004843 | -1.37 | Abscisic acid stress ripening2 |
| *Zm*00001d052591 | -1.35 | B3 domain-containing protein |
| *Zm*00001d027409 | -1.38 | B3 domain-containing transcription factor NGA2 |
| *Zm*00001d028815 | 2.41 | Pathogenesis-related protein 10 |
| *Zm*00001d028814 | 2.72 | Pathogenesis-related protein 10 |
| *Zm*00001d023811 | 3.65 | Pathogenesis-related protein STH-21 |
| *Zm*00001d028816 | 5.46 | Pathogenesis-related protein 10 |
| *Zm*00001d007188 | -1.22 | Ethylene insensitive 3-like 2 protein |
| *Zm*00001d003451 | -1.47 | Ethylene insensitive 3-like 5 protein |
| *Zm*00001d003924 | -2.02 | Glycerophosphodiester phosphodiesterase GDPD2 |

**Fig. S1.** Regression analysis between qRT-PCR and RNA-seq for the 4 selected genes, data were log10 transformed.


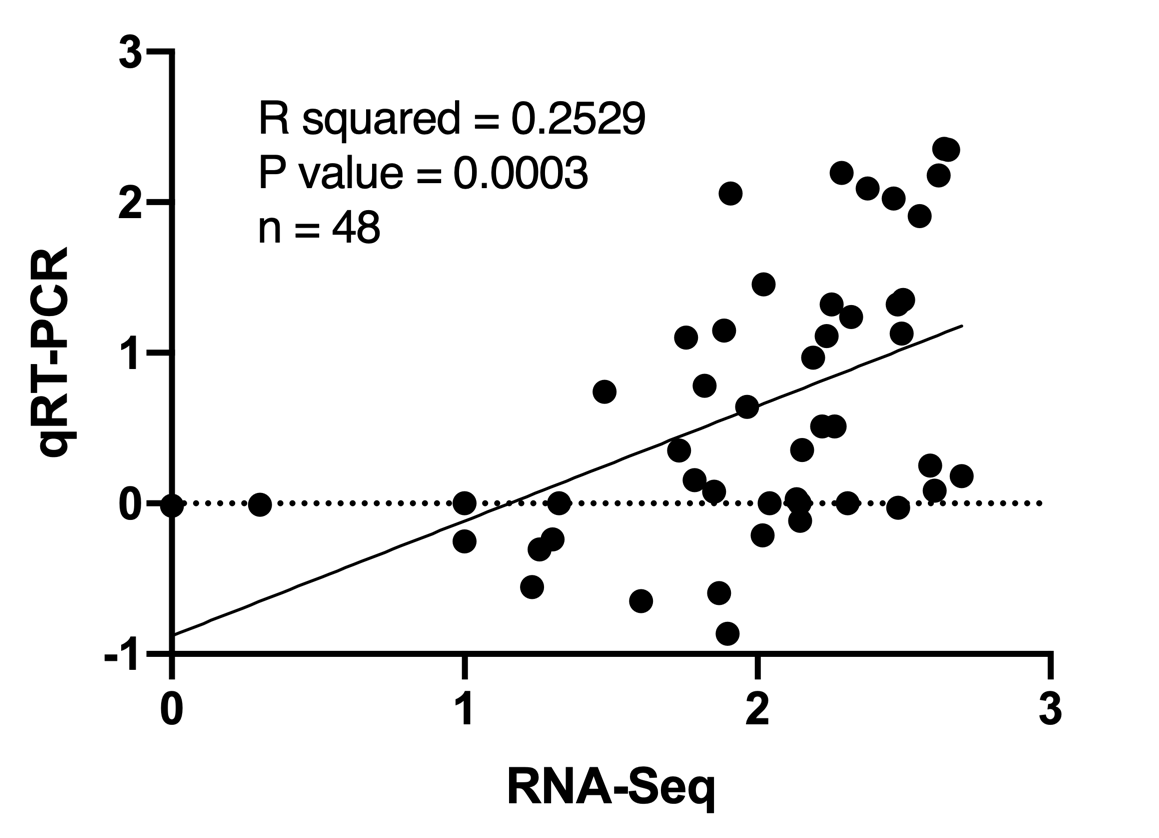


**Fig. S1**
